# *COBL* is a novel hotspot for *IKZF1* deletions in childhood acute lymphoblastic leukemia

**DOI:** 10.18632/oncotarget.10590

**Published:** 2016-07-13

**Authors:** Bruno Almeida Lopes, Claus Meyer, Thayana Conceição Barbosa, Udo zur Stadt, Martin Horstmann, Nicola C. Venn, Susan Heatley, Deborah L. White, Rosemary Sutton, Maria S. Pombo-de-Oliveira, Rolf Marschalek, Mariana Emerenciano

**Affiliations:** ^1^ Pediatric Hematology-Oncology Program, Research Center, Instituto Nacional de Câncer, Rio de Janeiro, RJ, Brazil; ^2^ Diagnostic Center of Acute Leukemia/Institute of Pharmaceutical Biology/ZAFES, Goethe-University of Frankfurt, Biocenter, Germany; ^3^ Center for Diagnostics, University Medical Center Hamburg Eppendorf, Hamburg, Germany; ^4^ Research Institute Children's Cancer Center, Hamburg, Germany; ^5^ Department of Pediatric Hematology and Oncology, University Medical Center Hamburg-Eppendorf, Hamburg, Germany; ^6^ Children's Cancer Institute, Lowy Cancer Research Centre UNSW, Sydney, New South Wales, Australia; ^7^ South Australian Health and Medical Research Institute (SAHMRI), Adelaide, South Australia, Australia; ^8^ Discipline of Medicine, University of Adelaide, Adelaide, South Australia, Australia

**Keywords:** acute lymphoblastic leukemia, COBL, IKZF1, RAG, relapse

## Abstract

*IKZF1* deletion (Δ*IKZF1*) is an important predictor of relapse in childhood B-cell precursor acute lymphoblastic leukemia. Because of its clinical importance, we previously mapped breakpoints of intragenic deletions and developed a multiplex PCR assay to detect recurrent intragenic Δ*IKZF1*. Since the multiplex PCR was not able to detect complete deletions (*IKZF1* Δ1-8), which account for ~30% of all Δ*IKZF1*, we aimed at investigating the genomic scenery of *IKZF1* Δ1-8. Six samples of cases with *IKZF1* Δ1-8 were analyzed by microarray assay, which identified monosomy 7, isochromosome 7q, and large interstitial deletions presenting breakpoints within *COBL* gene. Then, we established a multiplex ligation-probe amplification (MLPA) assay and screened copy number alterations within chromosome 7 in 43 diagnostic samples with *IKZF1* Δ1-8. Our results revealed that monosomy and large interstitial deletions within chromosome 7 are the main causes of *IKZF1* Δ1-8. Detailed analysis using long distance inverse PCR showed that six patients (16%) had large interstitial deletions starting within intronic regions of *COBL* at diagnosis, which is ~611 Kb downstream of *IKZF1,* suggesting that *COBL* is a hotspot for Δ*IKZF1*. We also investigated a series of 25 intragenic deletions (Δ2–8, Δ3–8 or Δ4–8) and 24 relapsed samples, and found one *IKZF1-COBL* tail-to-tail fusion, thus supporting that *COBL* is a novel hotspot for Δ*IKZF1*. Finally, using RIC score methodology, we show that breakpoint sequences of *IKZF1* Δ1-8 are not analog to RAG-recognition sites, suggesting a different mechanism of error promotion than that suggested for intragenic Δ*IKZF1*.

## INTRODUCTION

Several genetic mutations are usually necessary for the onset of cancer [1]. In case of childhood acute lymphoblastic leukemia (ALL) only few mutations are required [2]. In most cases, products deriving from chromosomal translocations are the oncogenic initiating or driving lesions and have been characterized at the molecular and functional level during the last decades. In addition to the widely known gene fusions, genomic technologies - SNP arrays or whole genome sequencing - also allowed the identification of complementing genetic alterations that are important contributors of hematological malignancies. One of the identified alterations concerns the *IKZF1* gene, located at 7p12, encoding the transcription factor Ikaros, that is essentially involved in the development of the B-cell lineage [3].

The biology of *IKZF1* is complex because this gene consists of 8 exons, and encodes 11 different splice variants [4]. Five of these isoforms are translated into proteins that are acting as transcriptional activators (1, 2, 2a, 3, 3a), while six isoforms result in dominant-negative versions of Ikaros (4, 4a, 5, 6, 7, 8). The Ikaros protein composes a regulatory complex involving hematopoietic transcription factors (E2A, EBF1-3, and PAX5) with the distinct function of driving lymphoid development [5], which is impaired by “dominant-negative” isoforms.

In 2007, the first study demonstrating the importance of *IKZF1* deletions (Δ*IKZF1*) in leukemia was published [2]. Genome wide SNP array analyses of 242 patients revealed that several genes involved in B-lineage development are mutated in B-cell precursor ALL (BCP-ALL) patients. These initial findings were confirmed by a contemporary study that investigated 40 leukemia patients [6], which identified again recurrent submicroscopic deletions in several genes linked to B-lineage development (*PAX5, EBF1, TCF3, IKZF1* and others). Thereafter, clinical studies revealed that leukemia patients with Δ*IKZF1* should be classified as high-risk, as the presence of a deletion was shown to be an independent prognostic factor for event-free survival in pediatric BCP-ALL [7]. Of note, a subsequent international study showed that all types of Δ*IKZF1* are associated with unfavorable prognosis [8]. The clinical effects of Δ*IKZF1* and the results from basic research have been thoroughly reviewed [9].

Due to the clinical importance of Δ*IKZF1*, multiplex ligation-probe amplification (MLPA) assays have been developed to detect such alterations in BCP-ALL. As Δ*IKZF1* are predictive of relapse and given the lack of sensitivity of MLPA assays, scientific efforts focused on novel diagnostic tools for minimal residual disease investigation. In 2011, a real-time quantitative PCR assay was developed to detect the most common microdeletion in ALL *IKZF1*Δ3-6 (now known as *IKZF1*Δ4-7), which results in the Ik6 transcript [10]. This was followed in 2013 by two studies that developed multiplex PCR assays to detect recurrent intragenic deletions of *IKZF1*, such as Δ2-3, Δ2-7, Δ2-8, Δ4-7, and Δ4-8 [11, 12]. Although these methodologies present a higher sensitivity of at least 10^-2^, they are not able to detect complete deletions (*IKZF1* Δ1-8), which represent ~30% of all deletions. Therefore, the aim of this study was to unravel the genomic landscape of *IKZF1* Δ1-8.

## RESULTS

### Clinical and genetic characteristics of BCP-ALL with *IKZF1* complete deletions

Patients with *IKZF1* Δ1-8 were mainly male (60.5%), aged 1-9 years old (72.1%) at diagnosis, and only 30.2% had WBC ≥ 50 × 10^9^/L. The predominant immunophenotype was c-ALL (65.1%).

Copy number alterations (CNAs) in genes frequently altered in BCP-ALL were compared between patients with or without *IKZF1* deletions in order to discover possible concomitant alterations associated with *IKZF1* Δ1-8. The results showed that *IKZF1* Δ1-8 deletions extended to its surrounding genes, such as *ZPBP* (deleted in 85.7% of cases), *FIGNL1* (90.5%) and *DDC* (90.5%). On the other hand, deletions of *EBF1* and *BTG1* were respectively absent or rarely (4.1%) identified in patients with *IKZF1* Δ1-8 (Supplementary Table S1).

### Genome-wide CNAs in samples with complete deletion of *IKZF1* at diagnosis

**Figure 1:**
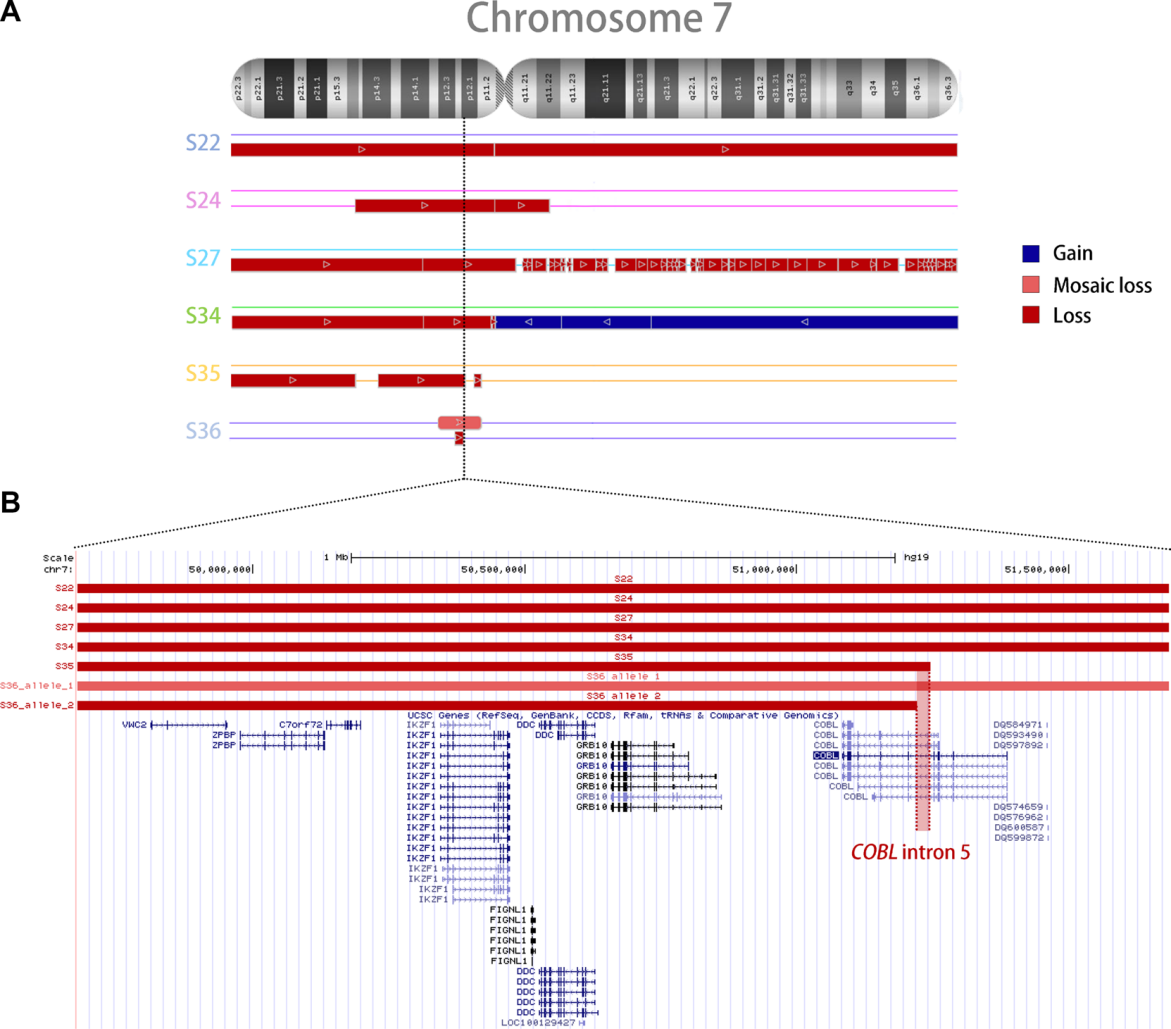
Copy number alterations in the chromosome 7 of six samples (S22, S24, S27, S34, S35, and S36) with complete deletion of *IKZF1* (**A**) The figure shows chromosome 7 CNAs, where red and blue lines indicate deletions and amplifications, respectively. (**B**) UCSC Genome Browser closer view of the region containing *IKZF1* and its surrounding genes, showing complete deletions of *IKZF1* and breakpoint within *COBL* intron 5 in two samples (S35 and S36). Genomic coordinates were standardized to the GRCh37 (hg19) assembly of the human genome.

Six random DNA samples of BCP-ALL with *IKZF1* Δ1-8 were selected for the microarray assay. The details of each sample are presented in Supplementary Table S2. Microarray analysis revealed that samples with *IKZF1* Δ1-8 presented large deletions within chromosome 7 (*n* = 5) and partial loss of 9p arm (*n* = 3), as shown in Supplementary Figure S1. Three sorts of alterations on chromosome 7 were found for samples with *IKZF1* Δ1-8: monosomy 7 (*n* = 2), large interstitial deletions (*n* = 3), and isochromosome 7q (*n* = 1) (Figure 1A). Interestingly, two patients (S35 and S36) with large interstitial deletions presented breakpoints within *COBL* intron 5 (Figure 1B) downstream of *IKZF1*. On the other hand, there was no indication of a genetic hotspot upstream of *IKZF1,* as 50% of cases presented loss of chromosome 7 short arm, and the remaining patients had remarkably variable breakpoints at the 7p telomeric side.

### Screening CNAs on chromosome 7 with the customized MLPA

Because the microarray data showed that a diverse spectrum of alterations within chromosome 7 promotes *IKZF1* Δ1-8, we designed two customized MLPAs to identify such CNAs in the whole series of patients included in this study. The MLPA probes were distributed throughout chromosome 7, and most of them were placed within *COBL*. The main characteristics and localization of the MLPA probes are described in Supplementary Table S4, and probe sequences are available upon request.

First, the custom MLPAs were validated with five samples previously analyzed by microarray. The comparison of the results produced by both techniques is illustrated in Supplementary Figure S2. In sum, the MLPA results were concordant with microarray experiments. As expected, patient S22 (with monosomy 7 by microarray) presented monoallelic deletion for all of the probes tested on chromosome 7, while patients S35 and S36 (with *COBL* intron 5 rearrangements) presented deletion of a series of probes within *COBL*, thus correctly indicating the breakpoint was localizing within intron 5. Although our MLPA did not detect all of the expected CNAs (e.g. patient S24 bears an interstitial deletion within chromosome 7 and patient S34 has isochromosome 7q), the analysis of whole probe set contributed to the correct interpretation of results. Thus, we confirmed that our custom MLPAs were able to detect distinct CNAs within chromosome 7.

**Figure 2:**
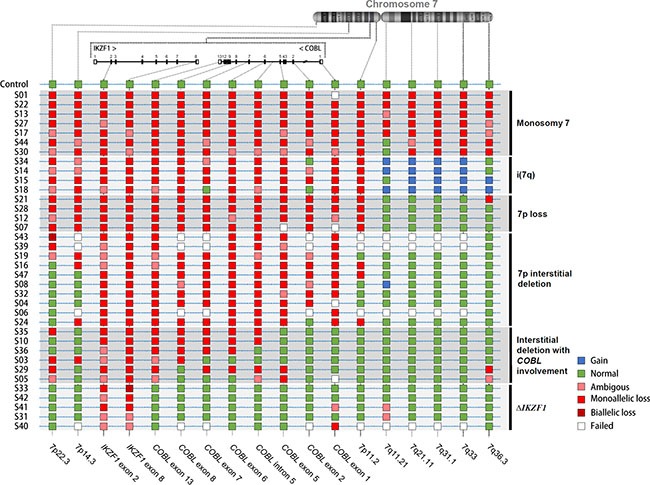
Chromosome 7 CNAs of pediatric BCP-ALL with *IKZF1 Δ*1-8 The CNA results for samples with *IKZF1* Δ1-8 are displayed in each row, while vertical lines specify the localization of MLPA probes within chromosome 7. The colored squares represent regions with amplification (blue), without CNAs (green), and monoallelic deleted (red) or biallelic deletions (dark red). Ambiguous results (pink square) represents borderline peak ratios. The samples were clustered into six groups based on CNAs for chromosome 7: monosomy 7, isochromosome (7q), 7p loss, 7p interstitial deletion, interstitial deletions in the chromosome 7, and complete deletion of *IKZF1* only.

Subsequently, the custom MLPAs were used to investigate CNAs for chromosome 7 in 43 BCP-ALL pediatric patients with *IKZF1* Δ1-8. The MLPA results could not be interpreted for seven samples, which were excluded from further analysis. Five sorts of alterations were identified on chromosome 7: monosomy 7 (*n* = 7), isochromosome 7q (*n* = 4), 7p loss (*n* = 4), large interstitial deletions within the 7p arm (*n* = 10), *IKZF1* Δ1-8 with breakpoints within *COBL* (*n* = 6), and *IKZF1* complete deletion without involvement of surrounding regions (*n* = 5) (Figure 2).

### Breakpoint sequences of *IKZF1* Δ1-8

We designed four long-distance inverse PCRs (LDI-PCRs) as well as two multiplexed long-distance PCRs (MP-PCRs) assays in order to find the sequence of the breakpoints predicted by both microarray and MLPA results. The sequences of breakpoints for *COBL* rearrangements are summarized in Supplementary Figure S3. Patient S10 presented a large interstitial deletion on the short arm of chromosome 7, comprising the whole *IKZF1* until *COBL* intron 5 and a subsequent ~1.1 Mb inversion. Patients S35 and S36 presented interstitial deletions of 18.8 and 1.7 Mb within 7p arm, respectively. The deletions fused *COBL* intron 5 to non-coding regions of the short arm of chromosome 7: 7p14.3-*COBL* (S35) and 7p12-*COBL* (S36).

### Characteristics of patients with breakpoints in *COBL*

*COBL* rearrangements were found in six patients with *IKZF1* Δ1-8 using our customized MLPA and LDI-PCR. We also investigated *IKZF1-COBL* fusions in 25 newly diagnosed ALL samples with *IKZF1* 3′-end deletions (i.e. retention of *IKZF1* exon 1 and a heterozygous deletion from exons 2, 3 or 4 through to exon 8). None of these 25 samples had *IKZF1-COBL* fusions. In addition, we detected a single patient with *IKZF1-COBL* fusion when RNA sequencing was performed on 24 relapsed BCP-ALL samples. Our initial MLPA analysis did not find this deletion, but closer inspection showed ratios of 0.84–0.93 for exons 4–8, consistent with 15–30% of cells in the sample having both *IKZF1-COBL* and *CDNK2A* microdeletions. This patient had an mRNA fusion between *IKZF1* exon 3 and a cryptic exon located in intron 5 of the *COBL* gene, resulting in an arbitrary fusion protein (Supplementary Figure S4). We successfully sequenced the breakpoints of this patient and three others (S10, S35, S36, and S48) (Supplementary Figure S3). Due to PCR size limitations, the breakpoint region of the remaining three samples with *COBL* rearrangements by MLPA (S03, S05, and S29) could not be confirmed by Sanger sequencing. Patient and laboratory characteristics of cases with *COBL*-rearrangements as well as their clinical data are described in Table 1.

**Table 1:** Demographic, laboratory and clinical characteristics of *IKZF1* deleted cases with *COBL* involvement

Characteristics	Patient identification						
	S03	S05	S10	S29	S35	S36	S48
Age (yrs)	1^†^	5	5	16	1^§^	5	15
Sex	M	F	M	M	F	F	M
Laboratory							
WBC (×10^9^/L)	5.0	5.7	16.4	55.0	459.6	7.5	NA
Immunophenotype	c-ALL	c-ALL	Pre-B	c-ALL	c-ALL	c-ALL	Pre-B
ALL subtype	NA	NA	NA	High hyperdiploid	*ETV6-RUNX1*	*ETV6-RUNX1*	B-other
*CDKN2A/B* status	Deleted	wt	NA	Deleted	Deleted	wt	Deleted
*PAX5* status	Deleted	Deleted	NA	Deleted	Deleted	wt	wt
Clinical data							
Clinical trial	COALL97	COALL92	COALL97	GBTLI-93	None^¶^	GBTLI-93	UKALLR3
CNS disease	No	No	No	No	NA	No	No
MRD (D33)	Negative	Negative	Negative	NA	-	NA	2E-2
CR (D33)	Yes	Yes	Yes	NA	-	NA	Yes
NCI risk group	LR	LR	LR	HR	HR	LR	HR
Relapse	NA	Yes	Yes	Yes	No	No	Yes
Time to relapse (yrs)	NA	6.5	9.0	2	-	-	3.3
Outcome	NA	Alive	Alive	Dead	Dead	Alive	Dead
Follow-up (mo)	NA	96	47	13	0.5	69	8

c-ALL, common acute lymphoblastic leukemia; CNS, central nervous system; CR, complete morphological remission; F, female; HR, high risk; LR, low risk; M, male; MRD, minimal residual disease; mo, months; NA, not available; NCI, national cancer institute; SR, standard risk; wt, wild-type; yrs, years.

† 13 months-old at diagnosis.

§ 20 months-old at diagnosis.

¶ The child died before any chemotherapy treatment.

### Identification of possible mechanisms underlying the deletions occurrence

**Figure 3:**
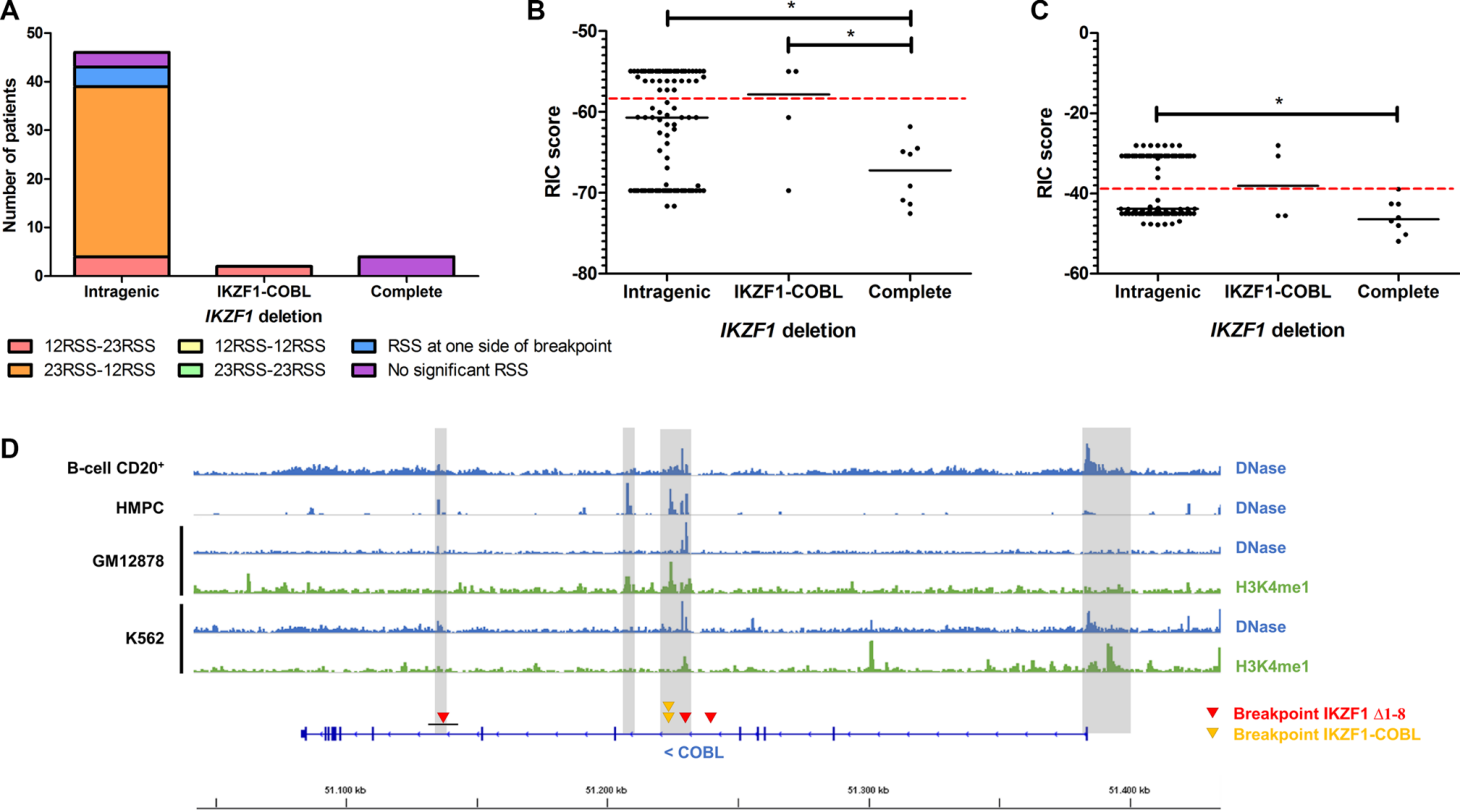
Comparison of RSSs at the breakpoint sequences of intragenic and complete deletions of *IKZF1* in childhood BCP-ALL (**A**) The figure illustrates the RSS combination at each side of the breakpoints for intragenic and complete deletions of *IKZF1*, showing that intragenic deletions, which includes *IKZF1*-*COBL* fusions, present 23RSS-12RSS and 12RSS-23RSS at the breakpoint region, while complete deletions of *IKZF1* do not present RAG analogous sequences. We also compared RAG-analogue sequences for (**B**) 12-spaced RSSs, and (**C**) 23-spaced RSSs using the RIC score methodology. The greater RIC scores indicates higher similarity to RSSs. Dashed red lines show threshold scores associated to functional RSSs, and dots represent RSS-analogous sequences for each patient. Because recombination events generally occurs between 12-RSS and 23-RSS, each side of breakpoint region is limited to one statistically significant and another sequence not significant for 12/23-RSSs. Intragenic deletions were collected from our previous study [12], and complete deletions from the current study. The results show that complete deletions of *IKZF1* do not present statistically significant RSS-analogous sequences (* *p-value* < 0.05). (**D**) Integrative Genomics Viewer (IGV) visualization of DNase-seq and ChIP-seq (target: H3K4me1) data retrieved from ENCODE database showing accessible chromatin sites within *COBL* for human primary CD20-positive B-cells, hematopoietic precursor-cells (HMPC), as well as B lymphoblastoid (GM12878) and chronic myeloid leukemia (K562) cell lines. The grey squares highlight the regions associated to open chromatin at the promoter, as well as intron 5 and intron 7 of *COBL* gene, located on the antisense strand of 7p12.1. Colored triangles illustrate the position of the breakpoints within *COBL* leading to *IKZF1* complete deletions (red) and *IKZF1-COBL* fusions (yellow). The black horizontal line at *COBL* intron 7 defines the range of breakpoint region for patient S03 detected by MLPA. The scale bar refers to human GRCh37/hg19 genome assembly.

After sequencing the breakpoints, we explored which mechanism would be involved in the generation of the interstitial deletions and gene fusions found in this study. First, we investigated RAG1/2 recombination signal sequences (RSSs) nearby the breakpoints. However, this analysis showed that most of the breakpoint sequences had RIC scores for 12RSSs and 23RSSs below the critical threshold (-38.81 and -58.45, respectively), and were not associated with functional RSSs. Only the patient S48 with *IKZF1-COBL* fusion presented significant RSS sequences at the proximity of the breakpoint site. Then, we compared the RIC scores of complete deletions and intragenic deletions of *IKZF1* from our previous report [12]. As summarized in the Figure 3A–3C, RSSs and RIC scores were significantly different between these groups. Intragenic deletions, including *IKZF1-COBL* fusions, presented RAG analog sequences, while complete deletions did not present functional RSSs, supporting the hypothesis that another mechanism may be associated with complete deletions of *IKZF1*. Moreover, data analyses from publicly available tracks from the ENCODE consortium showed that the active chromatin regions associated with the presence H3K4me1 and DNase hypersensitive sites were located at the promoter region, as well as intron 5 and intron 7 of *COBL* gene (Figure 3D).

## DISCUSSION

Nearly 30% of all Δ*IKZF1* in pediatric BCP-ALL comprise complete deletions of *IKZF1,* termed *IKZF1* Δ1-8. Here, we developed novel methods to characterize these patient cases in more detail. Our results showed that most of the *IKZF1* Δ1-8 derive from large interstitial deletions within chromosome 7, spanning genes either upstream (*ZPBP* and *C7orf72*, ~210 Kb upstream of *IKZF1*) or downstream (*FIGNL1* and *DDC*, 39 and 53 Kb downstream, respectively) of *IKZF1*. Interestingly, *EBF1* and *BTG1* deletions were found in patients with intragenic *IKZF1* deletions, but they were rarely deleted in the cohort of *IKZF1* Δ1-8 patients, corroborating with a previous report showing that *BTG1* and *EBF1* deletions co-occur in ALL [13]. Our data suggest that *IKZF1* Δ1-8 promotes the development of a leukemogenic process that is independent of alterations in *EBF1* and *BTG1*. Furthermore, a recent study found that mutual *BTG1* and *IKZF1* deletions cooperatively increased the incidence of relapse in pediatric BCP-ALL cases. However, the patients were not stratified based on *IKZF1* deletion subgroups [14]. Therefore, *BTG1* deletions may play a synergistic role with intragenic *IKZF1* deletions, but the same might not be true for *IKZF1* Δ1-8.

Contrary to intragenic deletions, *IKZF1* Δ1-8 is characterized by larger chromosomal deletions on chromosome 7. After a first screening round with microarrays in order to identify CNAs within chromosome 7, we developed a novel MLPA assay and performed detailed analyses to better characterize such alterations. In this study, children with BCP-ALL presented *IKZF1* Δ1-8 due to monosomy 7 (7/36, 19%) or large interstitial deletions that occurred on chromosome 7 (16/36, 44%). Alterations such as isochromosome 7q, 7p loss, and *IKZF1* Δ1-8 without involvement of surrounding regions were also found, but at much lower frequency. Earlier studies have also associated *IKZF1* Δ1-8 to monosomy 7 and interstitial deletions on chromosome 7 [11, 15, 16].

Our results also revealed seven patients with BCP-ALL bearing large interstitial deletions that all started within intronic regions of the *COBL*, which is localized ~611 Kb downstream of *IKZF1*. The frequency of *COBL* rearrangements varied for complete (16.7%) and intragenic (0%) deletions of *IKZF1* at diagnosis, and were found in 4.2% of cases at relapse. COBL is an actin nucleator and contains three copies of the WH2 (WASP homology 2) actin-binding domain, thus promoting actin polymerization [17]. It has important roles for neuronal morphogenesis and regulation of microvillar length. Its alterations have been associated to neuronal disorders (autism spectrum disorders) [18], and autoimmune diseases (eg. type 1 diabetes) [19]. In 2013, Meyer *et al*. characterized Δ*IKZF1* in pediatric BCP-ALL patients, and described for the first time an *IKZF1-COBL* tail-to-tail fusion as a consequence of an ~800 kb interstitial deletion between *IKZF1* intron 1 and *COBL* intron 5 [12]. In the present study, we also found an *IKZF1-COBL* fusion in a relapsed sample. In 2015, Baughn *et al*. found one patient with BCP-ALL and normal karyotype that presented a ~917 Kb interstitial deletion within chromosome 7, leading to *IKZF1* Δ1-8. Again, the breakpoint was located within *COBL* intron 6 [20]. In 2011, Flach *et al.* described one patient with an evolution from myelodysplastic syndrome to acute myeloid leukemia after accumulation of 7p12.1–12.2 deletion ranging from *IKZF1* to *COBL* [21]. In 2016, Duployez *et* al. reported a patient with myeloproliferative neoplasm who progressed to blast crisis upon acquisition of biallelic *IKZF1* deletions, as well as *EBF1* and *CDKN2A/B* deletions. In this case, *IKZF1* deletion involved its surrounding genes, from *VWC2* until *COBL* [22]. Furthermore, Gonzalez-Gonzalez *et al*. used a SNP-array to identify a ~941 Kb amplification between *IKZF1* and *COBL* intron 2 in a patient with metastatic colorectal cancer [23]. These data show that *COBL* rearrangements are recurrently found in *IKZF1* Δ1-8, and are also found in cases with intragenic deletions of *IKZF1* (*IKZF1-COBL* fusions), suggesting a relationship between genes located at 7p12.1 (*IKZF1*, *DDC*, *GRB10*, and *COBL*) and cancer. Interestingly, breakpoints within *COBL* were also found in autism spectrum disorders [18]. Although our data supported by these aforementioned studies suggest that *COBL* is a downstream hotspot for Δ*IKZF1*, the breakpoint sites varied considerably at the telomeric side of deletions. This finding has been concordant in both SNP array and MLPA screenings. For that reason, it was not possible to investigate any breakpoint hotspot upstream of *IKZF1* and, consequently, it is not feasible to include detection of *IKZF1* Δ1-8 in the multiplex PCR panels previously published.

Several studies have suggested that RAG-recognition errors might promote intragenic Δ*IKZF1*, based on the identification of RSS-analogue sequences in the vicinity of the identified breakpoints [24, 25]. However, the association between RAG recombination and *IKZF1* Δ1-8 is still unclear. Using the RIC score methodology, we have demonstrated that breakpoint sequences of *IKZF1* Δ1-8 investigated were not similar to RSSs. Also, *IKZF1* Δ1-8 with *COBL* rearrangements did not present additional nucleotides at the breakpoint sites, suggesting that complete deletions of *IKZF1* are not attributed to neither aberrant RAG activity nor terminal deoxynucleotidyl transferase (TdT) involvement. On the other hand, RSSs analogous sequences and additional nucleotides were found for patients with intragenic deletions of *IKZF1*, including patients with *IKZF1-COBL*. Therefore, analyses of breakpoint sequences reveal that intragenic deletions are possibly mediated by RAG-recombination events, while the remaining large interstitial deletions and monosomy 7 leading to *IKZF1* Δ1-8 are the result of other mechanism that caused chromosome instability. Such mechanisms could involve genomic hotspots due to the changes in the architecture of chromosomes, or, other mechanisms that are associated with DNA double-strand breakage. It is noteworthy that the breakpoints found in our study positioned within accessible chromatin regions, therefore, it is plausible that such area is more susceptible to double-strand DNA breaks.

In conclusion, we demonstrate that monosomy 7 and large interstitial deletions within chromosome 7 are the main causes of complete deletions of *IKZF1*. *COBL* rearrangements were recurrently found in these patients, showing that *COBL* represents a genetic hotspot for Δ*IKZF1*. Both cases with *IKZF1-COBL* had breakpoints within one base pair in *COBL* intron 5, so screening of new patient sets with 3′-*IKZF1* deletions may reveal similar patients with rare RAG initiated deletions. Further investigation of *COBL* rearrangements are needed to better characterize its role in BCP-ALL, and to answer the question whether the deletion of *COBL* or other genes localizing between *IKZF1* and *COBL* could be important for leukemogenesis and prognosis. For this purpose, we developed a customized MLPA assay for the evaluation of CNAs within *COBL*.

## MATERIALS AND METHODS

### Subjects

First, forty-three diagnostic samples of children with *IKZF1* Δ1-8 were selected, being 24 patients enrolled in a Brazilian previously published study [26] and 19 patients registered in the German CoALL [12]. Briefly, *IKZF1* Δ1-8 were analyzed by MLPA (SALSA MLPA P335-A3-B2 probe mix and/or SALSA MLPA P202-B1, MRC Holland, Amsterdam, The Netherlands), according to the manufacturer's recommendations. Based on the data obtained in this first screening, we also investigated two additional series of patients: (i) with lack of *IKZF1* exon 8, namely Δ2–8, Δ3–8 or Δ4–8 (*n* = 25), being 13 Brazilian and 12 Australian samples (ANZCHOG ALL8 or AIEOP-BFM ALL2009 trials), which were identified by MLPA analysis of 399 and 568 new diagnosis samples, respectively, and (ii) 24 relapse samples investigated by both MLPA and sequencing analysis. In accordance with the Declaration of Helsinki, clinical data collection (e.g. gender, age at diagnosis, white blood cell (WBC) count at diagnosis, and ALL subtype) and laboratory procedures have been evaluated and approved by the Ethics Committees of Instituto Nacional de Câncer-INCA (#33243214.7.0000.5274) and the Sydney Children's Hospital Network LNR.13.SCHN.367.

### Microarray assay

The microarray analysis was performed using the CytoScan HD Array according to the manufacturer protocol (Affymetrix. Inc., Santa Clara, CA, USA). Briefly, 250 ng of genomic DNA from six patients were digested with *Nsp*I, and then amplified with Titanium Taq PCR Kit (Clontech Laboratories, Inc., Mountain View, CA). After fragmentation and labeling, the DNA was hybridized to the microarray for 16 hours, washed on the GeneChip Fluidics Station 450, stained with Affymetrix GeneChip Stain Reagents, and scanned on the GeneChip Scanner 3000 7G (Affymetrix. Inc., Santa Clara, CA, USA). Data were analyzed using Chromosome Analysis Suite software version 3.0 (Affymetrix. Inc., Santa Clara, CA, USA) based on the GRCh37/hg19 build of the Human Genome Assembly.

### Multiplexed long-distance PCR

The breakpoints of interstitial deletions or gene fusion indicated by microarray were confirmed by MP-PCRs in order to analyze the breakpoints at the nucleotide level. The reaction was performed with a set of ten primers flanking a region of ~20 Kb surrounding each breakpoint. Amplification was performed with PCR Extender System (5Prime, Germany) and the primers listed in Supplementary Table S3.

### Customized multiplex ligation-dependent probe amplification

Two in-house customized MLPA assays were designed to investigate CNAs within chromosome 7, with a special focus on *COBL*. The design of the probes was based on the manual “Designing synthetic MLPA probes”, version 14 (MRC-Holland); probe details are described in Supplementary Table S4. The validation was performed by a comparison of CNA data between microarray and customized MLPA. In brief, 100 ng of genomic DNA were denatured and hybridized overnight with the customized probes. Then, the probes were ligated and amplified with SALSA MLPA EK1 reagents (MRC Holland, The Netherlands). The fragments were separated by ABI 3,500 Genetic Analyzer (Applied Biosystems, EUA), and analyzed with GeneMarker v1.85 (SoftGenetics), where the relative copy numbers are normalized according to the peaks observed in controls.

### Long distance inverse PCR

The LDI-PCR was used to analyze breakpoints within *COBL* intron 5. The technique was previously described for *KMT2A* rearrangements detection, and basically consists of seven steps: (1) DNA digestion with restriction enzymes, (2) religation of the ends to form circular DNA, (3) amplification of the circular DNA of interest (4) Agarose gel electrophoresis to separate derivative bands from wild type bands (5) gel extraction of derivative bands (6) sequencing of derivative bands (7) via BLAST alignment of the identified sequence with the human genome [27]. The primer sequences are listed in Supplementary Table S3.

### Identification of mechanisms leading to *IKZF1* deletions

RAG1/2 RSSs were investigated along the breakpoint sequences. The “RSS database” searches RSS sequences, consisting of a heptamer (5′-CACAGTG-3′) and a nonamer (5′-ACAAAAACC-3′) separated by either 12 or 23 nucleotides (12RSS and 23RSS), and classifies the sequences based on the “RIC score”, which estimates the similarity between the sequence of interest and the RSS consensus sequence [28]. 12RSSs and 23RSSs greater than -38.81 and -58.45, respectively, were attributed as possibly functional. In addition, DNase-seq and ChIP-seq data were retrieved from ENCODE and visualized with Integrative Genomics Viewer (IGV) version 2.3.77 to assess chromatin structure of *COBL*.

### Statistical analysis

This study compared clinical-demographic characteristics and CNAs between samples according to *IKZF1* status (*IKZF1* Δ1-8 vs. wild-type or intragenic deletions) with Fisher's exact test using SPSS Statistics 18 (IBM, EUA). For the analysis of RAG consensus sequences at breakpoints, we used an unpaired t test to compare RIC scores among *IKZF1* deletion subgroups. GraphPad Prism 5 (GraphPad Software, Inc., California, USA) software) was used for this analysis. *P*-values < 0.05 were interpreted as statistically significant.

## SUPPLEMENTARY MATERIAL FIGURES AND TABLES






