# Supplementary material for: COBL is a novel hotspot for IKZF1 deletions in childhood acute lymphoblastic leukemia
Source: Oncotarget. 2016 Jul 13;7(33):53064–73. doi: 10.18632/oncotarget.10590 (PMC5288169; doi:10.18632/oncotarget.10590)
Supplement: Supplementary file 1 [file oncotarget-07-53064-s001.pdf]

COBL is a novel hotspot for *IKZF1* deletions in childhood acute lymphoblastic leukemia

Supplementary Materials

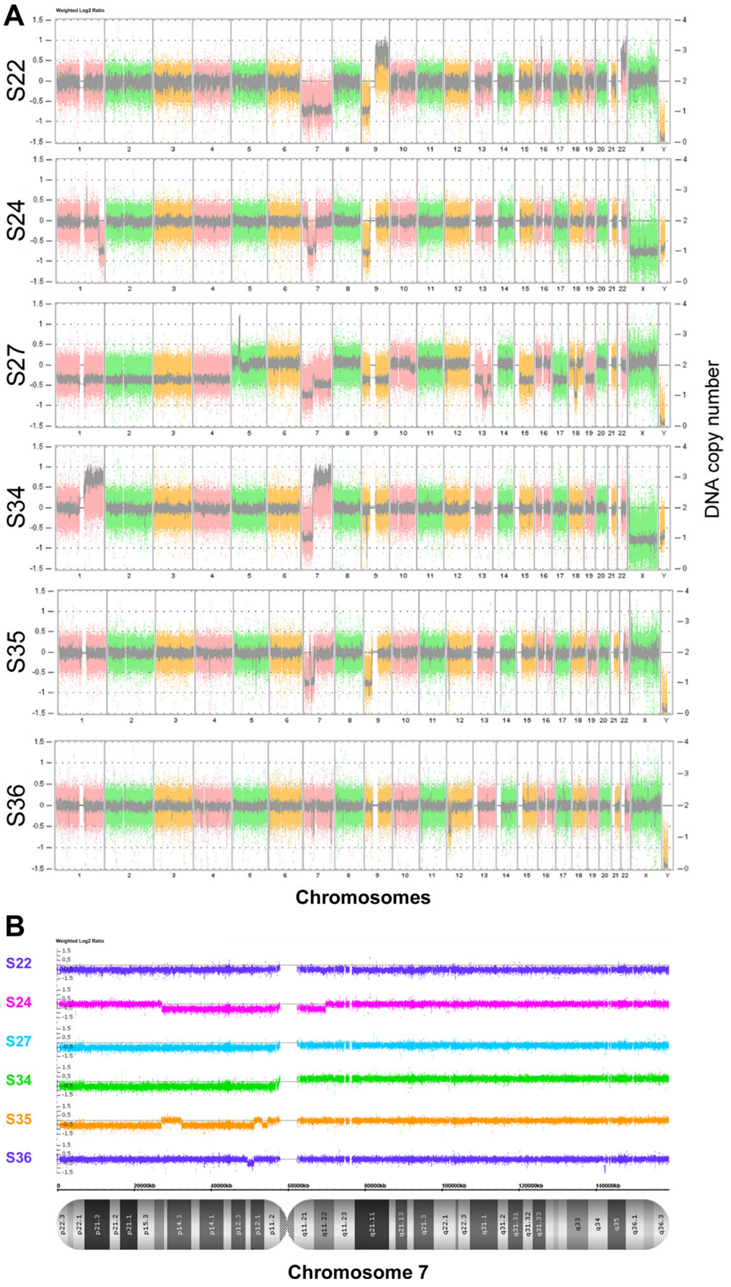

Supplementary Figure S1: Genomic CNAs of BCP-ALL samples with *IKZF1* Δ1-8. (**A**) The samples (S22, S24, S27, S34, S35, and S36) are identified on the left. The right axis illustrates the DNA copy number, while the horizontal axis shows the chromosome number. (**B**) Copy number ratio plot focused on chromosome 7. The graphic image was obtained from the analysis with Affymetrix Chromosome Analysis Suite (ChAS) 2.1. The entire chromosome 7 is shown, with the relative physical position indicated in kb based on the GRCh37/hg19 build of the Human Genome Assembly. A $\log_2$ copy number ratio of 0 indicates copy number $2n$, 1 indicates copy number $2n + 1$, and -1 indicates copy number $2n - 1$. The dashed line highlights the commonly deleted 7p12.1 region (*IKZF1*).

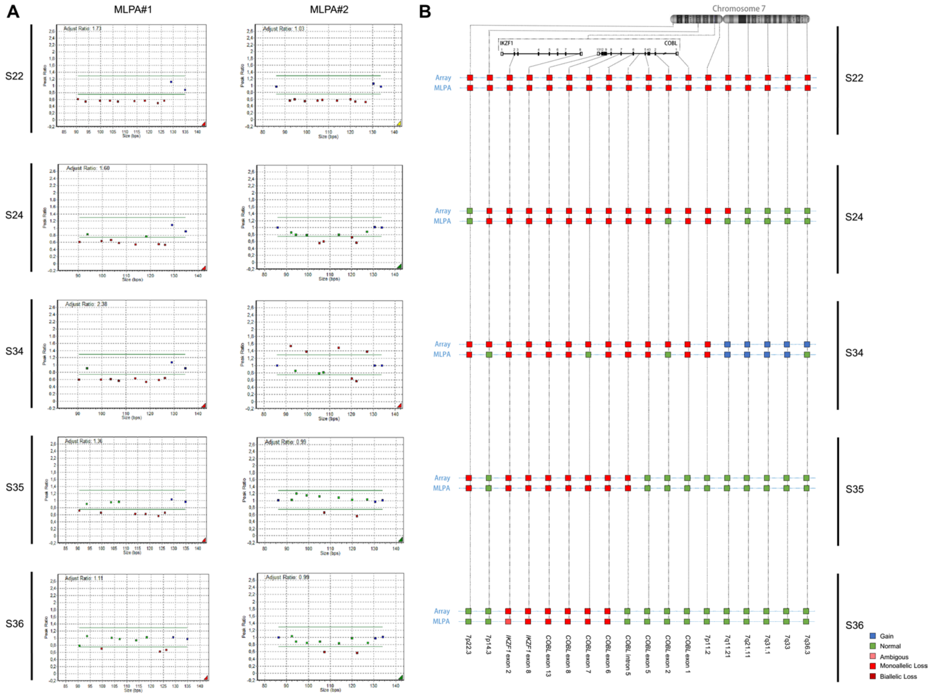

Supplementary Figure S2: Validation test of the custom MLPAs to investigate CNAs on chromosome 7. (**A**) Two MLPA probesets, MLPA#1 (left) and MLPA#2 (right), were used to investigate CNAs on chromosome 7. The validation test included five samples, which are displayed in each row. The horizontal bars of the graphs represents the thresholds for either gains (upper line) or losses (lower line). Green dots correspond to normal copy numbers, while red dots define CNAs. The blue dots illustrates control probes. (**B**) The illustration translates the MLPA validation results. The vertical lines indicate the localization of MLPA probes on chromosome 7, *IKZF1*, and *COBL*, which are illustrated at the top and written at the bottom. The CNA results of both CytoScan HD array and MLPA assay are illustrated in the rows for each sample. The colors of each square indicate the DNA copy number status, such as amplified (blue), wild-type (green), and deleted (red).

Patient S10: 7p12.1 – *COBL* intron 5

```
12RSS                                                                          CACAGTG   - 12 -   ACAAAAACC (RIC: -50.24)
                                                                                || |                       |
7p12.1 ATTTGCTGATCTCCCTCTCCTAGCTTACCCCTGAATCCCTTTTCCTTCCATTGTTCCTATCTCACTGCAATGCCATATCCCCATTGCTCAGTTTCTGCCCTTGGCAGTGATC
       ||||||||||||||||||||||||||||||||||||||||||||||||||||||||
S10    ATTTGCTGATCTCCCTCTCCTAGCTTACCCCTGAATCCCTTTTCCTTCCATTGTTCTTGCAAATTAACTCAGGTACCTTGTCTCATCCGTTAGCACAGTTTATCACTGCTGT
                                                               ||||||||||||||||||||||||||||||||||||||||||||||||||||||||
COBL   CATTTGCACTTTATCAGACTGATATAATAATTTCTTTTCCATTTTTGTGTGCTGAATTGCAAATTAACTCAGGTACCTTGTCTCATCCGTTAGCACAGTTTATCACTGCTGT
                                                   ||| |||                       ||  ||
23RSS                                           GGTTTTTGT      -    23    -     CACTGTG (RIC: -61.82)
```

Patient S35: 7p14.3 – *COBL* intron 5

```
23RSS      (RIC: -71.42) GGTTTTTGT      -    23    -     CACTGTG
                         |                                |   ||
7p14.3 GACTCCATCAAAAAAAAAGAAAAGAAAAAGAGAAGTCTGCCAGTCAAGAATATCATGCCCAGCAAAGCTATCCTTCAGAAATGAAGGAGAAATAAAATCTTTCACAGACAAG
       |||||||||||||||||||||||||||||||||||||||||||||||||||||||||
S35    GACTCCATCAAAAAAAAAGAAAAGAAAAAGAGAAGTCTGCCAGTCAAGAATATCATGTCTTTCATCTTATTAATGTCTTTTCAAGAAAAATTTACTTTCCATGTTTAACAAT
                                                                |||||||||||||||||||||||||||||||||||||||||||||||||||||||
COBL   CTTTTCTTATCAGTAAATCACGTTCAAACACAATGGATACATGAAATTTTTTAATTGTCTTTCATCTTATTAATGTCTTTTCAAGAAAAATTTACTTTCCATGTTTAACAAT
                |   |              |||  ||
12RSS         GGTTTTTGT   - 12 -   CACTGTG (RIC: -42.60)
```

Patient S36: 7p12 – *COBL* intron 5

```
12RSS                                                                    (RIC: -45.97) CACAGTG   - 12 -   ACAAAAACC
                                                                                       |||| |              || |
7p12   TATACTATACTATACTATACTATACTATACTATAATACCATTATCCTATACCAAATAACACCATATAATACCATTATACCACACTATACCAAATAACACCATACTATACCA
       ||||||||||||||||||||||||||||||||||||||||||||||||||||||||
S36    TATACTATACTATACTATACTATACTATACTATAATACCATTATCCTATACCAAATCCGGTGAGAAGCTCATAGCATAAAAAAAAGATACTTTTTTATTAGCACATCACTG
                                                               |||||||||||||||||||||||||||||||||||||||||||||||||||||||
COBL   TGTGGGTCCCAGCTGCCATGGGGGATTGTAAAGATCTTATCAAATGTGTGCACACTCCGGTGAGAAGCTCATAGCATAAAAAAAAGATACTTTTTTATTAGCACATCACTG
                           ||   ||||             | ||||
12RSS                      GGTTTTTGT   - 12 -   CACTGTG (RIC: -38.93)
```

Patient S48: *IKZF1* intron 3 – *COBL* intron 5

```
12RSS                                                      CACAGTG   - 12 -   ACAAAAACC (RIC: -30.62*)
                                                           |||||||            || ||||
IKZF1  AATAATCTGAATTGACGGCATCCAGGGATCTCAGAAATTATTAGTACATCCCACAGTGAATTACCACCTTACTAAAATATTCATGGGTATATACTATGGATTTGTTTTAT
       |||||||||||||||||||||||||||||||||||||||||||||||||||||
S48    AATAATCTGAATTGACGGCATCCAGGGATCTCAGAAATTATTAGTACATCCCGGCCCTATCCAAGTCAGTAAACCTAAACAGAAAAACGTTATCTGCTCAGGACTATTTA
                                                                 |||||||||||||||||||||||||||||||||||||||||||||||||||||
COBL   AATGAAGGAACACTCAGAAGCTCTACACCTATGGGGTAGTGTGTGTTAAACAGAGTGTATCCAAGTCAGTAAACCTAAACAGAAAAACGTTATCTGCTCAGGACTATTTA
                                                          || ||||                       | ||||||
23RSS                                                     CACAGTG   -      23       -   ACAAAAACC (RIC: -54.99*)
```

Patient 3: *IKZF1* intron 1 – *COBL* intron 5

```
12RSS                                                             CACAGTG   - 12 -   ACAAAAACC (RIC: -28.01*)
                                                                  ||| ||             ||  |||
IKZF1  TGTACATTTTTGATCTAGGTCTTAGAAACGTAGAGTTTCAGAGGATCAGCATTATACACACTGTCACACACACACACACTTAAAATTCAGATGAGGAACAAGATAGGAAT
       |||||||||||||||||||||||||||||||||||||||||||||||||||
P#3    TGTACATTTTTGATCTAGGTCTTAGAAACGTAGAGTTTCAGAGGATCAGCACTCGGACCCTATCCAAGTCAGTAAACCTAAACAGAAAAACGTTATCTGCTCAGGACTAT
                                                                    ||||||||||||||||||||||||||||||||||||||||||||||||||
COBL   ATAAATGAAGGAACACTCAGAAGCTCTACACCTATGGGGTAGTGTGTGTTAAACAGAGTGTATCCAAGTCAGTAAACCTAAACAGAAAAACGTTATCTGCTCAGGACTAT
                                                             || ||||                       | ||||||
23RSS                                                       CACAGTG    -      23       -   ACAAAAACC (RIC: -54.99*)
```

Supplementary Figure S3: Breakpoint sequences of *IKZF1* Δ1-8 and *IKZF1-COBL* samples. The figure illustrates the sequence of breakpoint regions in samples with *IKZF1* Δ1-8 generated by large deletions from *COBL*. The sequence of the samples are flanked by the wild-type sequences of the corresponding regions. RAG recombination signal sequences (RSS12 and RSS23) are highlighted in blue, followed by RIC score values; (*). indicates a good correlation between RIC score and RSS functionality.

```
IKZF1  1      MDADEGQDMSQVSGKESPPVSDTPDEGDEPMPIPEDLSTTSGGQQSSKSDRVVASNVKVETQSDEENGRACEMNGEECAEDLRMLDASGEKMNGSHRDQG
              |||||||||||||||||||||||||||||||||||||||||||||||||||||
S48    1      MDADEGQDMSQVSGKESPPVSDTPDEGDEPMPIPEDLSTTSGGQQSSKSDRVVGPIVMVLTEGSLHHQ*-------------------------------

IKZF1  101    SSALSGVGGIRLPNGKLKCDICGIICIGPNVLMVHKRSHTGERPFQCNQCGASFTQKGNLLRHIKLHSGEKPFKCHLCNYACRRRDALTGHLRTHSVGKP
S48    101    ----------------------------------------------------------------------------------------------------

IKZF1  201    HKCGYCGRSYKQRSSLEEHKERCHNYLESMGLPGTLYPVIKEETNHSEMAEDLCKIGSERSLVLDRLASNVAKRKSSMPQKFLGDKGLSDTPYDSSASYE
S48    201    ----------------------------------------------------------------------------------------------------

IKZF1  301    KENEMMKSHVMDQAINNAINYLGAESLRPLVQTPPGGSEVVPVISPMYQLHKPLAEGTPRSNHSAQDSAVENLLLLSKAKLVPSEREASPSNSCQDSTDT
S48    301    ----------------------------------------------------------------------------------------------------

IKZF1  401    ESNNEEQRSGLIYLTNHIAPHARNGLSLKEEHRAYDLLRAASENSQDALRVVSTSGEQMKVYKCEHCRVLFLDHVMYTIHMGCHGFRDPFECNMCGYHSQ
S48    401    ----------------------------------------------------------------------------------------------------

IKZF1  501    DRYEFSSHITRGEHRFHMS*--------------------------------------------------------------------------------
S48    501    ----------------------------------------------------------------------------------------------------
```

Supplementary Figure S4: Comparison of amino acid residue sequences for both the wild-type isoform of *IKZF1* and the *IKZF1* truncated protein for the sample S48. Zinc-finger domains of *IKZF1* are highlighted in blue. No domains were identified for the truncated protein.

Supplementary Table S1: Frequency of copy number alterations in Brazilian pediatric BCP-ALL subsequent cases according to *IKZF1* status. See Supplementary_Table_S1

Supplementary Table S2: Clinical characteristics of patients included in the microarray analysis

ID	Gender	WBC[a]	Blasts (%)	Age (yrs)	Immunophenotype	ALL subtype[b]
S22	Female	110.0	60	13	c-ALL	*BCR-ABL1*
S24	Male	336.0	100	0.5[¶]	pro-B ALL	Other
S27	Male	58.1	55	1.1[§]	pro-B ALL	*KMT2A-r*
S34	Male	125.7	59	14	pro-B ALL	Other
S35	Female	459.6	80	1.7[†]	c-ALL	*ETV6-RUNX1*
S36	Female	7.5	50	5	c-ALL	*ETV6-RUNX1*

c-ALL, common acute lymphoblastic leukemia; ID, identification; WBC, White cell count.

[a]WBC × 10^9 leucocytes per liter of blood.

[b]Cytogenetic subgroup of BCP-ALL. The subgroup defined as “Other” was diploid and negative for the investigation of *ETV6-RUNX1, TCF3-PBX1, KMT2A*-r, and *BCR-ABL1*.

[¶]6 months at diagnosis.

[§]13 months at diagnosis.

[†]20 months at diagnosis.

Supplementary Table S3: Description of the primers of multiplex long-distance PCR and long distance inverse PCR. See Supplementary_Table_S3

Supplementary Table S4: Description of the custom MLPA probe sets

		Custom MLPA#1			Custom MLPA#2		
#	Size[a]	Gene	Region	Band	Gene	Region	Band
1	**88**	*NINL*[¶]	Intron 21	20p11.21	NINL[¶]	Intron 21	20p11.21
2	**92**	*IKZF1*	Exon 2	7p12.2	GUSB	Exon 6	7q11.21
3	**96**	*VIPR2*	Exon 12	7q36.3	COBL	Exon 2	7p12.1
4	**101**	*COBL*	Exon 13	7p12.1	PCLO	Exon 6	7q21.11
5	**105**	*COBL*	Exon 1	7p12.1	PDE1C	Intron 18	7p14.3
6	**109**	*COBL*	Exon 5	7p12.1	COBL	Exon 7	7p12.1
7	**116**	*COBL*	Intron 5	7p12.1	LRRN3	Exon 4	7q31.1
8	**120**	*PDGFA*	Intron 5	7p22.3	CHCHD2	Intron 2	7p11.2
9	**124**	*COBL*	Exon 6	7p12.1	COBL	Exon 8	7p12.1
10	**128**	*IKZF1*	Exon 8	7p12.2	TMEM	Exon 2	7q33
11	**132**	*MCM4*[¶]	Exon 16	8q11.21	MCM4[¶]	Exon 16	8q11.21
12	**136**	*POP4*[¶]	Exon 1	19q12	POP4[¶]	Exon 1	19q12

[a]The size of the MLPA fragments is indicated in base pairs (pb).

[¶]Control probes.
