# Supplementary material for: COBL is a novel hotspot for IKZF1 deletions in childhood acute lymphoblastic leukemia
Source: Oncotarget. 2016 Jul 13;7(33):53064–73. doi: 10.18632/oncotarget.10590 (PMC5288169; doi:10.18632/oncotarget.10590)
Supplement: Supplementary file 2 [file oncotarget-07-53064-s002.doc]

**Table S1.** Frequency of copy number alterations in Brazilian pediatric BCP-ALL subsequent cases according to *IKZF1* status

| **Gene** | **Localization** |  | ***IKZF1* Status** | | |
| --- | --- | --- | --- | --- | --- |
|  |  |  | **Wild-type**  ***n* (%)** | **Intragenic deletion^a^**  ***n* (%)** | **Complete deletion^b^**  ***n* (%)** |
| **MLPA SALSA P335** | |  |  |  |  |
| *EBF1* | 5q33.3 |  |  |  |  |
| Wild-type |  |  | 264 (84.3) | 35 (56.5) | 22 (91.7) |
| Deleted |  |  | 16 (5.1) | 17 (27.4) | 0 (0.0) |
| Amplified |  |  | 33 (10.5) | 10 (16.1) | 2 (8.3) |
|  |  |  | *P* = 0.617 | ***P* = 0.002** | Ref. |
| *JAK2* | 9p24.1 |  |  |  |  |
| Wild-type |  |  | 69 (93.2) | 16 (94.1) | 2 (66.7) |
| Deleted |  |  | 3 (4.1) | 0 (0.0) | 1 (33.3) |
| Amplified |  |  | 2 (2.7) | 1 (5.9) | 0 (0.0) |
|  |  |  | *P* = 0.154 | *P* = 0.158 | Ref. |
| *CDKN2A* | 9p21.3 |  |  |  |  |
| Wild-type |  |  | 209 (66.8) | 31 (50.0) | 13 (54.2) |
| Deleted |  |  | 88 (28.1) | 28 (45.2) | 11 (45.8) |
| Amplified |  |  | 16 (5.1) | 3 (4.8) | 0 (0.0) |
|  |  |  | *P* = 0.110 | *P* = 1.000 | Ref. |
| *CDKN2B* | 9p21.3 |  |  |  |  |
| Wild-type |  |  | 221 (70.6) | 38 (61.3) | 13 (54.2) |
| Deleted |  |  | 74 (23.6) | 24 (38.7) | 10 (41.7) |
| Amplified |  |  | 18 (5.8) | 0 (0.0) | 1 (4.2) |
|  |  |  | *P* = 0.082 | *P* = 0.804 | Ref. |
| *PAX5* | 9p13.2 |  |  |  |  |
| Wild-type |  |  | 205 (65.5) | 20 (32.3) | 10 (41.7) |
| Deleted |  |  | 64 (20.4) | 36 (58.1) | 12 (50.0) |
| Amplified |  |  | 44 (14.1) | 6 (9.7) | 2 (8.3) |
|  |  |  | ***P* = 0.004** | *P* = 0.449 | Ref. |
| *ETV6* | 12p13.2 |  |  |  |  |
| Wild-type |  |  | 229 (73.2) | 38 (61.3) | 17 (70.8) |
| Deleted |  |  | 67 (21.4) | 21 (33.9) | 6 (25.0) |
| Amplified |  |  | 17 (5.4) | 3 (4.8) | 1 (4.2) |
|  |  |  | *P* = 0.797 | *P* = 0.448 | Ref. |
| *BTG1* | 12q21.33 |  |  |  |  |
| Wild-type |  |  | 263 (84.0) | 41 (66.1) | 22 (91.7) |
| Deleted |  |  | 29 (9.3) | 16 (25.8) | 1 (4.2) |
| Amplified |  |  | 21 (6.7) | 5 (8.1) | 1 (4.2) |
|  |  |  | *P* = 0.710 | ***P* = 0.018** | Ref. |
| *RB1* | 13q14.2 |  |  |  |  |
| Wild-type |  |  | 264 (84.3) | 43 (69.4) | 19 (79.2) |
| Deleted |  |  | 36 (11.5) | 11 (17.7) | 2 (8.3) |
| Amplified |  |  | 13(4.2) | 8 (12.9) | 3 (12.5) |
|  |  |  | *P* = 1.000 | *P* = 0.330 | Ref. |
| *SHOXAREA* | Xp22 |  |  |  |  |
| Wild-type |  |  | 240 (76.9) | 50 (80.6) | 21 (87.5) |
| Deleted |  |  | 4 (1.3) | 2 (3.2) | 0 (0.0) |
| Amplified |  |  | 68 (21.8) | 10 (16.1) | 3 (12.5) |
|  |  |  | *P* = 1.000 | *P* = 1.000 | Ref. |
| *CRLF2* | Xp22 |  |  |  |  |
| Wild-type |  |  | 257 (82.1) | 48 (77.4) | 19 (79.2) |
| Deleted |  |  | 10 (3.2) | 7 (11.3) | 2 (8.3) |
| Amplified |  |  | 46 (14.7) | 7 (11.3) | 3 (12.5) |
|  |  |  | *P* = 0.215 | *P* = 1.000 | Ref. |
| *CSF2RA* | Xp22 |  |  |  |  |
| Wild-type |  |  | 253 (80.8) | 52 (83.9) | 20 (83.3) |
| Deleted |  |  | 8 (2.6) | 4 (6.5) | 1 (4.2) |
| Amplified |  |  | 52 (16.6) | 6 (9.7) | 3 (12.5) |
|  |  |  | *P* = 0.507 | *P* = 1.000 | Ref. |
| *IL3RA* | Xp22 |  |  |  |  |
| Wild-type |  |  | 242 (77.3) | 48 (77.4) | 21 (87.5) |
| Deleted |  |  | 15 (4.8) | 9 (14.5) | 1 (4.2) |
| Amplified |  |  | 56 (17.9) | 5 (8.1) | 2 (8.3) |
|  |  |  | *P* = 1.000 | *P* = 0.268 | Ref. |
| *P2RY8* | Xp22 |  |  |  |  |
| Wild-type |  |  | 70 (76.1) | 16 (80.0) | 4 (100.0) |
| Deleted |  |  | 8 (8.7) | 3 (15.0) | 0 (0.0) |
| Amplified |  |  | 14 (15.2) | 1 (5.0) | 0 (0.0) |
|  |  |  | *P* = 1.000 | *P* = 1.000 | Ref. |
| **MLPA SALSA P202** | |  |  |  |  |
| *IKZF2* | 2q34 |  |  |  |  |
| Wild-type |  |  | 8 (72.7) | 15 (45.5) | 12 (57.1) |
| Deleted |  |  | 3 (25.0) | 15 (45.5) | 9 (42.9) |
| Amplified |  |  | 0 (0.0) | 3 (9.1) | 0 (0.0) |
|  |  |  | *P* = 0.465 | *P* = 0.777 | Ref. |
| *ZPBP* | 7p12.2 |  |  |  |  |
| Wild-type |  |  | 11 (100) | 31 (93.9) | 3 (14.3) |
| Deleted |  |  | 0 (0.0) | 2 (6.1) | 18 (85.7) |
| Amplified |  |  | 0 (0.0) | 0 (0.0) | 0 (0.0) |
|  |  |  | ***P* < 0.001** | ***P* < 0.001** | Ref. |
|  |  |  |  |  |  |
| *FIGNL1* | 7p12.1 |  |  |  |  |
| Wild-type |  |  | 11 (100) | 31 (93.9) | 2 (9.5) |
| Deleted |  |  | 0 (0.0) | 1 (3.0) | 19 (90.5) |
| Amplified |  |  | 0 (0.0) | 1 (3.0) | 0 (0.0) |
|  |  |  | ***P* < 0.001** | ***P* < 0.001** | Ref. |
|  |  |  |  |  |  |
| *DDC* | 7p12.1 |  |  |  |  |
| Wild-type |  |  | 11 (100) | 29 (87.9) | 2 (9.5) |
| Deleted |  |  | 0 (0.0) | 3 (9.1) | 19 (90.5) |
| Amplified |  |  | 0 (0.0) | 1 (3.0) | 0 (0.0) |
|  |  |  | ***P* < 0.001** | ***P* < 0.001** | Ref. |
|  |  |  |  |  |  |
| *MIR31* | 9p21.3 |  |  |  |  |
| Wild-type |  |  | 8 (72.7) | 21 (63.6) | 11 (52.4) |
| Deleted |  |  | 2 (18.2) | 12 (36.4) | 10 (47.6) |
| Amplified |  |  | 1 (9.1) | 0 (0.0) | 0 (0.0) |
|  |  |  | *P* = 0.240 | *P* = 0.571 | Ref. |
|  |  |  |  |  |  |
| *CEP170B* | 14q32.33 |  |  |  |  |
| Wild-type |  |  | 7 (63.6) | 25 (75.8) | 16 (76.2) |
| Deleted |  |  | 0 (0.0) | 4 (12.1) | 2 (9.5) |
| Amplified |  |  | 4 (36.4) | 4 (12.1) | 3 (14.3) |
|  |  |  | *P* = 1.000 | *P* = 1.000 | Ref. |
| *MTA1* | 14q32.33 |  |  |  |  |
| Wild-type |  |  | 7 (63.6) | 25 (75.8) | 13 (61.9) |
| Deleted |  |  | 1 (9.1) | 5 (15.2) | 3 (14.3) |
| Amplified |  |  | 3 (27.3) | 3 (9.1) | 5 (25.8) |
|  |  |  | *P* = 1.000 | *P* = 1.000 | Ref. |
| *IGHD* | 14q32.33 |  |  |  |  |
| Wild-type |  |  | 6 (54.5) | 27 (81.8) | 15 (71.4) |
| Deleted |  |  | 2 (18.2) | 5 (15.2) | 5 (23.8) |
| Amplified |  |  | 3 (27.3) | 1 (3.0) | 1 (4.8) |
|  |  |  | *P* = 1.000 | *P* = 0.480 | Ref. |
| *IKZF3* | 17q12 |  |  |  |  |
| Wild-type |  |  | 9 (81.8) | 27 (81.8) | 14 (66.7) |
| Deleted |  |  | 1 (9.1) | 5 (15.2) | 7 (33.3) |
| Amplified |  |  | 1 (9.1) | 1 (3.0) | 0 (0.0) |
|  |  |  | *P* = 0.222 | *P* = 0.183 | Ref. |

^a^ Intragenic deletion refers to any deletion limited to *IKZF1*

^b^ Complete *IKZF1* deletion refers to ∆1-8 deletion

*P* - values refer to the comparison between wild-type versus deleted cases, considering cases with *IKZF1* complete deletion as the reference group.
