# Supplementary material for: COBL is a novel hotspot for IKZF1 deletions in childhood acute lymphoblastic leukemia
Source: Oncotarget. 2016 Jul 13;7(33):53064–73. doi: 10.18632/oncotarget.10590 (PMC5288169; doi:10.18632/oncotarget.10590)
Supplement: Supplementary file 3 [file oncotarget-07-53064-s003.doc]

**Table S3. Description of the primers of multiplex long-distance PCR and long distance inverse PCR.**

| **Method** | **Primers** | **Sequence (5’ - 3’)** |
| --- | --- | --- |
| MP-PCR for S35 | 7p14.3.F1 | AATCCTATCAATTCCTCCCCATGTGTCAAG |
|  | 7p14.3.F2 | CTATCAATGGCCCTTCCAAGCTATCAGGTA |
|  | 7p14.3.F3 | GATTGCAGATTTCTGGGAGAATAAGGACGA |
|  | 7p14.3.F4 | CAGGCAAATTGGAAGATGAGTAGGAAGGAG |
|  | 7p14.3.F5 | CAACAAGAAGAAAGGACCAAGGCAACAAAT |
|  | 7p14.3.F6 | CCAAACTGGTCCTCTCTGAGTGATATGGTG |
|  | 7p14.3.F7 | GGAATCACAAAGACACAATAGCCAAAGCAA |
|  | 7p14.3.F8 | ACAGGAAACCAAACACCACATGTTCTCACT |
|  | 7p14.3.F9 | TTCGAAGGACATCATCAAGAAAATGAATGG |
|  | 7p14.3.F10 | TGCAGCAATAGTCCAGAACAGATGTTCAAA |
|  | COBL.I5.R1 | CAAAAGGAGTAATCTTGGGTGATGGTGGTT |
|  | COBL.I5.R2 | CCAGTTCACAGTAAAATCCACCATTCCTCA |
|  | COBL.I5.R3 | GCCCACCATAGTGTACTTTACCCAATTGCT |
|  | COBL.I5.R4 | CCTTTAAGCCATGCAAAATTGTTGTTCCTT |
|  | COBL.I5.R5 | AGTTATGAAAGCAATGCCTGGAGAACCAAC |
|  | COBL.I5.R6 | TAGCGTATTCCCTAGGGTCTCAGCTTCTCA |
|  | COBL.I5.R7 | TCTGCAGAGGTCTCAGTGAACAGATGCTAA |
|  | COBL.I5.R8 | AAGGATTTCTGAAAACTACCTGTGCCCTCA |
|  | COBL.I5.R9 | GAATGGAGTATCCATCCCCTCAAGCATTTA |
|  | COBL.I5.R10 | TGCTTTGGTTGCCTATACTTGTGGGGTACT |
| MP-PCR for S36 | 7p12.2.F1 | ATAGCCTCCAATTCTTCCACTTCGACTCCT |
|  | 7p12.2.F2 | GCCCAGCCTCATATTCTTTCACTTAATCCA |
|  | 7p12.2.F3 | GACCCATGTCCTGAAGGAGCAATGAAGTAT |
|  | 7p12.2.F4 | AATGCATGAAGAGACTAACATGCGGAGACT |
|  | 7p12.2.F5 | AGACATTGGAATGGCCTGAGATACTCCACT |
|  | 7p12.2.F6 | CACAAAATTACTCCTTTGGGGTCTTGCAGT |
|  | 7p12.2.F7 | GGTAGGAGGTAGACACGAAGGAAGCTAGGG |
|  | 7p12.2.F8 | TGTTTTCTTCGTTTCCTAGCCCCTTCTACC |
|  | 7p12.2.F9 | AGCACACAGATAAGGCCCTCAAGTGAATTT |
|  | 7p12.2.F10 | CATGCCTGGCCAGCTCTATTTCTTTAAGACT |
|  | COBL.I5.R11 | GTGTCCTCAGAATCAAGTTAGCAGCCTTCA |
|  | COBL.I5.R12 | TATAATCATGCAGATCTTCCAACCCCACCT |
|  | COBL.I5.R13 | TCGAGCTTTATAACGACCTCGTGCACATAC |
|  | COBL.I5.R14 | TTCTTAGACAATCGCAGAACCTCTGTGGAC |
|  | COBL.I5.R15 | GGAATGGCCCTATCGTACCGGTAACTTTTA |
|  | COBL.I5.R16 | CTTTCTGGGCAGATACCAGTGGTCGTTATT |
|  | COBL.I5.R17 | AGAAGGCAGAAAAGCAAGAGAGCATGGTAG |
|  | COBL.I5.R18 | AGTGGAAGACACAGCAAACTCAGCTCTCAT |
|  | COBL.I5.R19 | AGAAGAGAAAGCAGAGGTGCACAAAGGAGT |
|  | COBL.I5.R20 | TGTAGAGGACAGACCATGGGAACTTCAAAA |
| LDI-PCR#1 | COBL.I5.F1 | TGCAAGGCAGTTACTAATGAAACTCCCAAAA |
|  | COBL.I5.R21 | AAATGCAAAGTGCTAGCTTTATGGTGGTGA |
|  | COBL.I5.R22 | GGCTTAGTGGGGACATGGTCAGTATTGATT |
|  | COBL.I5.R23 | TATGAGGATTAAATGAGTGCCAAGCACAGG |
|  | COBL.I5.R24 | AACACAACAACTGGCTCTCTTCTTCCTTCC |
|  | COBL.I5.R25 | AAACTCTGATTTGAGGTATTGGGGCAGATG |
|  | COBL.I5.R26 | CATGGAGATTGGCAGAAGTCAATGTTGATA |
|  | COBL.I5.R27 | TGGCTATCAGCAGTCATATTGCCCATCTAC |
| LDI-PCR#2 | COBL.I5.F2 | AGGGCTGAAGTTGACTTTATAGCCAGTGGA |
|  | COBL.I5.R3 | GCCCACCATAGTGTACTTTACCCAATTGCT |
|  | COBL.I5.R4 | CCTTTAAGCCATGCAAAATTGTTGTTCCTT |
|  | COBL.I5.R5 | AGTTATGAAAGCAATGCCTGGAGAACCAAC |
|  | COBL.I5.R6 | TAGCGTATTCCCTAGGGTCTCAGCTTCTCA |
| LDI-PCR#3 | COBL.I5.F3 | CTGAGACCCTAGGGAATACGCTAGACAGGA |
|  | COBL.I5.R28 | TTGTCTTTCTAAATTGCCTCTCTGCCATCA |
|  | COBL.I5.R29 | TCTGCAGAGGTCTCAGTGAACAGATGCTAA |
|  | COBL.I5.R30 | TGCTAAACTCTGAGCCTACGCTCTGTTCAC |
|  | COBL.I5.R31 | GTTCCCCACTACCTGTGCCATCCTTCTAC |
| LDI-PCR#4 | COBL.I5.F4 | CCCCACAAGTATAGGCAACCAAAGCAAATA |
|  | COBL.I5.R32 | GCACCTTTGTTGAAAATGACTTCGCTGTAA |
|  | COBL.I5.R33 | TGATCCTTTGAATTTCTGCGGTGTTAGTTG |
|  | COBL.I5.R34 | CTCCCTTCCTGTCTTCCTTTAGTGAAGGTG |

MP-PCR, multiplex long-distance PCR; LDI-PCR, long distance inverse PCR
